# OPTEM-oHCM: OPtimal Targeted Polymer-based EMbolization for Obstructive Hypertrophic CardioMyopathy

**DOI:** 10.1016/j.jaccas.2025.105859

**Published:** 2025-12-10

**Authors:** Evandro Martins Filho, Thiago Schumann Munhoz, Sidney Munhoz Júnior

**Affiliations:** a Department of Interventional Cardiology, Santa Casa de Misericórdia de Maceió, Maceió, Brazil; b Instituto Dante Pazzanese de Cardiologia, São Paulo, Brazil; c Laboratório de Hemodinâmica e Cardiologia Intervencionista do Centro-Oeste, Cuiabá, Brazil

**Keywords:** ethylene-vinyl alcohol, hypertrophic cardiomyopathy, left ventricular outflow tract obstruction, obstructive hypertrophic cardiomyopathy, polymer embolization, septal reduction therapy

## Abstract

**Background:**

Obstructive hypertrophic cardiomyopathy often requires septal reduction in symptomatic patients, but standard alcohol septal ablation may be limited in some cases.

**Case Summary:**

A 72-year-old woman presented with dyspnea and syncope. Investigations revealed dynamic left ventricular outflow tract gradient up to 167 mm Hg. Targeted embolization using ethylene-vinyl alcohol copolymer on septal and circumflex branches achieved complete gradient resolution.

**Discussion:**

This case demonstrates polymer-based embolization as a feasible alternative for complex cases, offering precise targeting beyond traditional septal branches, aligned with current guidelines for nonsurgical candidates.

**Take-Home Messages:**

Polymer embolization provides effective left ventricular outflow tract relief in obstructive hypertrophic cardiomyopathy with atypical supply. Functional balloon testing optimizes outcomes in high-risk patients.

## Visual Summary

**Figure:**
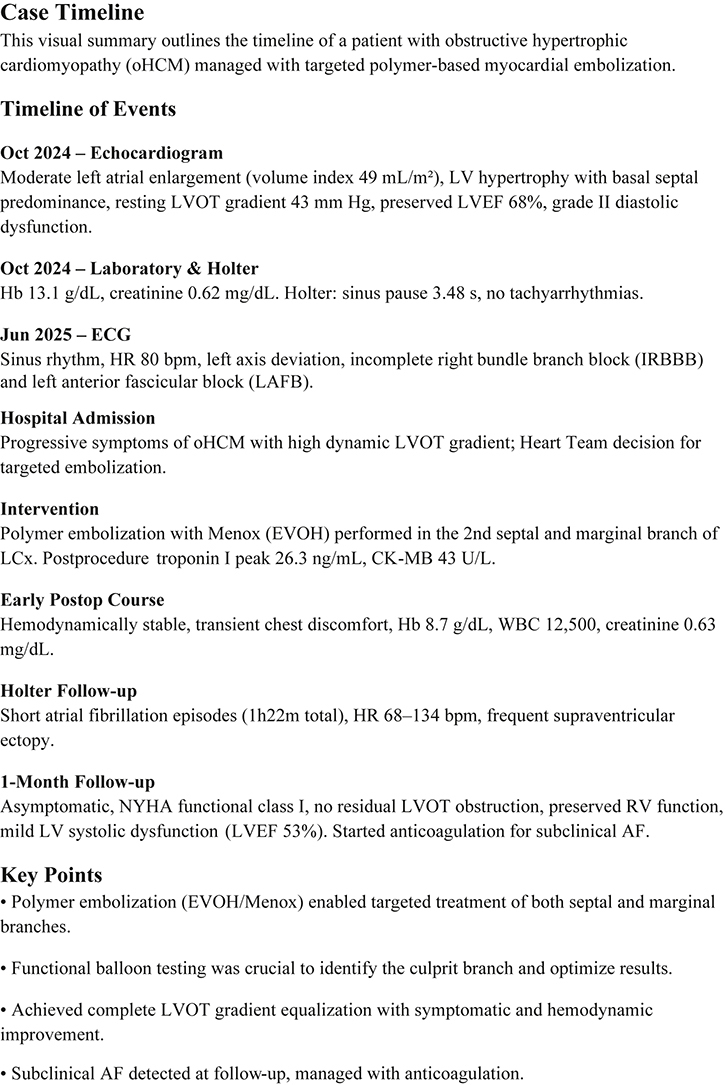

**Visual Summary:**
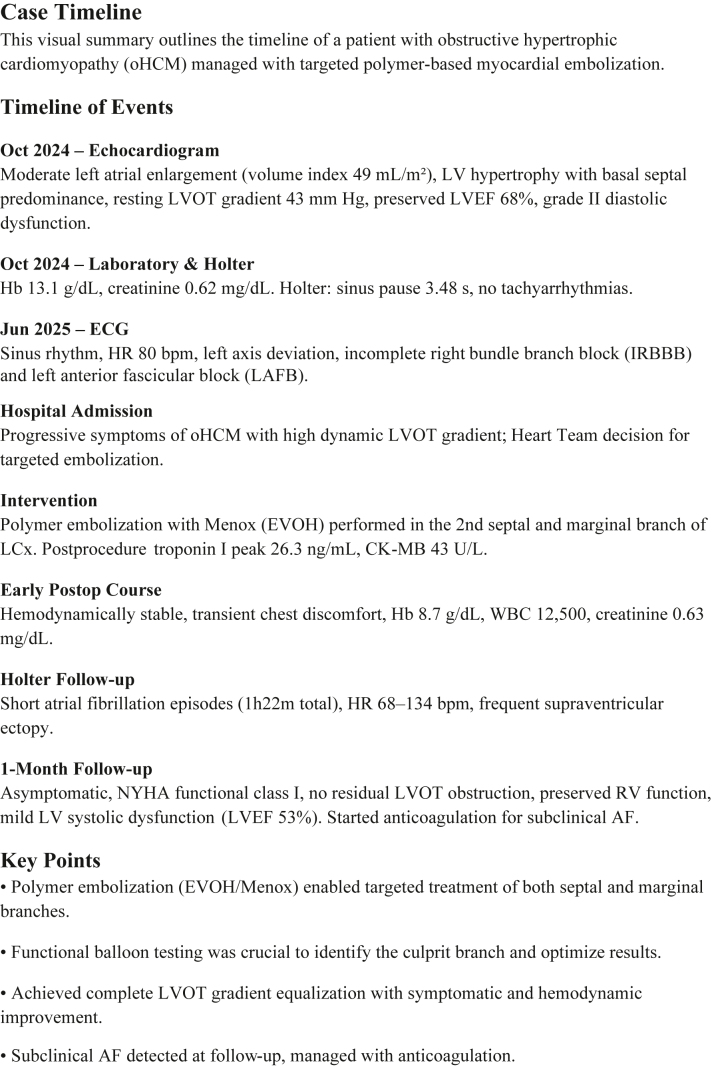
Targeted Polymer Embolization Resolves LVOT Obstruction in oHCM

## History of Presentation

A 72-year-old woman presented with progressive exertional dyspnea (NYHA functional class III), recurrent syncope, and dizziness. Her physical examination revealed a harsh systolic murmur at the left sternal border, which increased with Valsalva maneuver.

**Take-Home Messages**

• This case illustrates the utility of polymer-based embolization for obstructive hypertrophic cardiomyopathy in patients with nonstandard anatomy, achieving superior gradient reduction compared to traditional methods.
• Balloon occlusion testing under stress is crucial for identifying optimal targets and improving procedural outcomes.

## Past Medical History

Her medical history was notable for long-standing hypertension, type 2 diabetes mellitus, dyslipidemia, prior ischemic stroke with residual dysarthria, and chronic kidney disease (stage 3).

## Differential Diagnosis

In addition to obstructive hypertrophic cardiomyopathy (oHCM), differential considerations for her symptoms included aortic stenosis, ischemic heart disease, and arrhythmia-mediated syncope.

## Investigations

**Figure 1:**
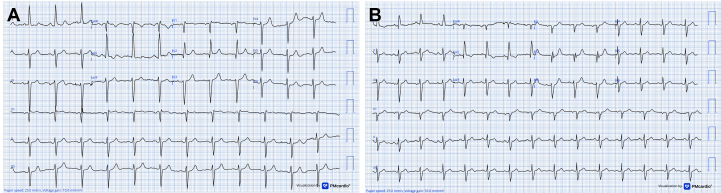
Serial Electrocardiograms Before and After Targeted Myocardial Embolization This figure compares baseline and postprocedural ECGs to evaluate conduction and repolarization changes following embolization in obstructive hypertrophic cardiomyopathy. (A) Baseline ECG with IRBBB, LAFB, and LVH with repolarization abnormalities. (B) Postembolization ECG with resolved conduction delays, persistent LVH, and new anteroseptal Q waves, without AV block. These findings confirm procedural safety regarding conduction system integrity while noting expected ischemic changes. AV = atrioventricular; ECG = electrocardiogram; IRBBB = incomplete right bundle branch block; LAFB = left anterior fascicular block; LVH = left ventricular hypertrophy.

Electrocardiography demonstrated left ventricular hypertrophy with incomplete right bundle branch block and left anterior fascicular block (Figure 1A). Ambulatory Holter monitoring documented a 3.48-second sinus pause without tachyarrhythmia. Transthoracic echocardiography showed preserved left ventricular ejection fraction (68%), moderate left atrial enlargement, and a resting left ventricular outflow tract (LVOT) gradient of 43 mm Hg.

Coronary computed tomography angiography revealed a right-dominant coronary system with no obstructive lesions and an Agatston calcium score of zero. Cardiac magnetic resonance imaging confirmed asymmetric basal and mid-septal hypertrophy (maximal wall thickness 15 mm) with systolic anterior motion of the mitral valve, moderate mitral regurgitation (MR), and patchy late gadolinium enhancement, consistent with reverse-curve hypertrophic cardiomyopathy.^1^^,^^2^

Invasive hemodynamic assessment demonstrated a resting LVOT gradient of 80 mm Hg, which increased dynamically to 167 mm Hg during dobutamine infusion (10 μg/kg/min).

## Management

**Figure 2:**
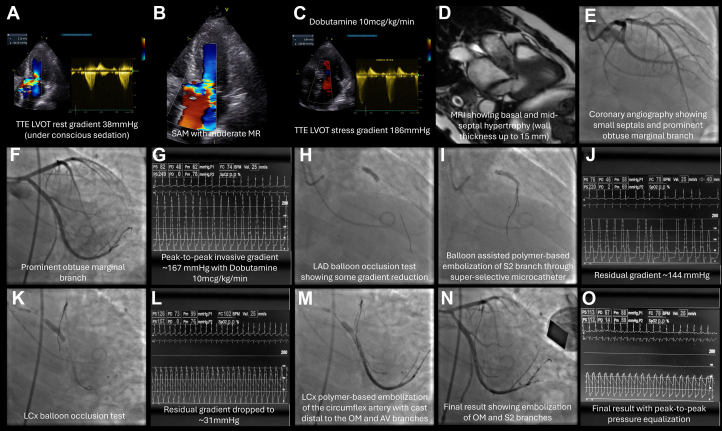
OPTEM-oHCM Stepwise Strategy This figure illustrates diagnostic and interventional steps in managing obstructive hypertrophic cardiomyopathy using optimized targeted embolization. (A-C) Echocardiography with resting (38 mm Hg) and dobutamine-induced (186 mm Hg) LVOT gradients, with SAM and MR. (D) Cardiac MRI with basal/mid-septal hypertrophy (up to 15 mm). (E and F) Coronary angiography with small septals and prominent OM branch. (G) An invasive ∼167 mm Hg gradient. (H and I) LAD balloon test and S2 embolization. (K-N) LCx testing and final embolization, with gradient resolution (O). LAD = left anterior descending; LCx = left circumflex; LVOT = left ventricular outflow tract; MR = mitral regurgitation; MRI = magnetic resonance imaging; OM = obtuse marginal; OPTEM-oHCM = optimized targeted embolization for obstructive hypertrophic cardiomyopathy; S2 = second septal perforator; SAM = systolic anterior motion.

The Heart Team elected to proceed with the OPTEM-oHCM (OPtimal Targeted Polymer-based EMbolization for Obstructive Hypertrophic CardioMyopathy) technique, employing an ethylene-vinyl alcohol (EVOH)-based liquid embolic system,^3^ given the patient's high surgical risk and the limited institutional experience with surgical myectomy. Under conscious sedation with intraprocedural transthoracic echocardiography (TTE) guidance, serial balloon occlusion tests using a 6.0 × 9-mm hypercompliant balloon, identified the second septal perforator (S2) and a marginal branch of the left circumflex artery (LCx) as contributors to the dynamic gradient (evaluated both by TTE and invasive hemodynamics tracings). Embolization of S2 (Figure 2I) yielded suboptimal reduction, with a residual gradient of 144 mm Hg (Figure 2J) under dobutamine stimulation (peak 167 mm Hg at baseline) (Figure 2G), as illustrated in Figure 2. Subsequent LCx balloon testing (Figure 2K) showed a marked drop in gradient to 31 mm Hg (Figure 2L).

Targeted embolization of the LCx marginal branch was performed (Figures 2M and 2N), carefully preserving the atrioventricular and obtuse marginal branches. A total of 3 mL of an EVOH copolymer (Menox, Meril Life Sciences) was administered in two 1.5-mL aliquots through a microcatheter under continuous fluoroscopic monitoring.

Final invasive hemodynamics showed complete LVOT gradient equalization (Figure 2O), and intraprocedural TTE revealed systolic anterior motion improvement (residual mild MR) and preserved LV function. Post embolization, the patient had transient chest pain resolved with opioids, peak troponin I of 26.3 ng/mL, and no rhythm disturbances (Figure 1B).

During the same hospitalization, 5 days after the embolization procedure, the patient underwent successful implantable cardioverter-defibrillator implantation for primary prevention of sudden cardiac death,^4^ based on European Society of Cardiology 2024 and American College of Cardiology/American Heart Association 2020 criteria.^5^

## Outcome and Follow-Up

At 1-month follow-up, the patient was asymptomatic (NYHA functional class I). Echocardiography demonstrated no residual LVOT obstruction, mild LV systolic dysfunction (LVEF 53%) with mild segmental wall motion abnormalities (inferior/inferolateral hypokinesia), mild MR/tricuspid regurgitation, and preserved right ventricular function. Subclinical paroxysmal atrial fibrillation was detected on Holter monitoring, prompting withdrawal of aspirin and initiation of apixaban 2.5 mg twice a day. No conduction abnormalities or other arrhythmias were observed during hospitalization or follow-up.

## Discussion

In patients with oHCM who remain symptomatic despite optimized medical therapy, septal reduction therapy is warranted.^5^ While surgical myectomy remains the gold standard,^6^^,^^7^ its availability is limited in many regions, including Brazil, underscoring the need for less invasive alternatives.

This case illustrates the feasibility of polymer-based embolization in a patient with atypical coronary anatomy, where the dominant driver of the LVOT gradient was a marginal branch of the circumflex artery. Standard alcohol septal ablation (ASA), which targets left anterior descending septal perforators,^8^ may not adequately address such nontraditional sources.

We employed an EVOH copolymer agent, originally developed for neurovascular interventions.^9^ EVOH formulations such as Onyx (Medtronic), Menox (Meril Life Sciences), Squid (Balt), and Phil (MicroVention/Terumo) provide high radiopacity, controlled delivery, and a lower risk of reflux or catheter adhesion compared with alcohol.^3^^,^^9^^,^^10^ Unlike ethanol, which diffuses unpredictably and causes broader myocardial necrosis,^7^^,^^8^ EVOH allows gradual, targeted embolization with potentially lower rates of atrioventricular block,^3^^,^^10^ although conduction disturbances can still occur. Careful patient selection and close monitoring are therefore essential.

Compared with ASA, where only ∼40% of patients achieve near-complete (≥90%) gradient reduction and up to 20% fail to reach a 50% decrease,^8^ EVOH embolization offers the possibility of more complete relief by enabling precise targeting of multiple or nonseptal branches. Importantly, residual LVOT gradient has been identified as an independent predictor of all-cause mortality, with a 1% increase in risk for every 1-mm Hg increment,^8^ emphasizing the value of achieving full hemodynamic resolution. EVOH's physical properties—viscosity, radiopacity, and nonadhesive delivery—facilitate this precision, potentially offsetting its higher cost through improved outcomes.^3^^,^^9^^,^^10^

In the present case, a total of 3 mL of Menox (Meril Life Sciences) was administered through a dimethyl sulfoxide-compatible microcatheter under continuous fluoroscopic guidance. Initial embolization of a septal branch yielded incomplete gradient reduction. Subsequent balloon occlusion testing revealed a dominant circumflex marginal branch, whose embolization resulted in complete hemodynamic resolution. This highlights the unique advantage of functional testing in guiding therapy.

Balloon occlusion provides dynamic, functional information on gradient behavior, whereas contrast echocardiography in ASA primarily defines perfused territory. In oHCM, where obstruction is dynamic and extends beyond septal hypertrophy, this distinction is critical. Gradients fluctuate with loading conditions, are often absent under sedation in the cathlab, and may vary spontaneously by over 200 mm Hg; such variability risks misclassification in up to 40% of patients if resting measurements alone are used. The addition of dobutamine enhances the reliability of balloon occlusion testing by replicating physiological stress and unmasking the true culprit branch.

Compared with ASA, polymer embolization expands the therapeutic scope beyond left anterior descending septals and may reduce conduction system injury.^3^^,^^10^ Compared with myectomy, it is less invasive, avoids general anesthesia, and can be performed in centers without surgical backup.^5^, ^6^, ^7^ Taken together, these features suggest that EVOH-based embolization represents a promising option for selected high-risk or anatomically complex patients. Prospective studies will be essential to confirm its long-term safety and efficacy.^3^^,^^5^^,^^10^

## Conclusions

Targeted EVOH embolization of a dominant marginal branch of the circumflex artery, following suboptimal gradient reduction with septal embolization, resulted in effective LVOT gradient relief in a fragile, nonsurgical candidate with high-risk oHCM and atypical myocardial supply. This approach represents a safe and effective alternative when conventional septal ablation is insufficient, or anatomy is nonstandard.

## Funding Support and Author Disclosures

The authors have reported that they have no relationships relevant to the contents of this paper to disclose.

## Appendix

Video 1 Fluoroscopic Demonstration of EVOH (Menox, Meril Life Sciences) Injection Into the LCx Marginal Branch, Performed Under Concomitant Balloon Inflation at the Level of Lateral Branches to Preserve Their Patency and Prevent Polymer Reflux This illustrates controlled embolization for targeted myocardial intervention in obstructive hypertrophic cardiomyopathy. EVOH = ethylene vinyl alcohol; LCx = left circumflex.

## References

[1] Maron B.J., Gardin J.M., Flack J.M., Gidding S.S., Kurosaki T.T., Bild D.E. (1995). Prevalence of hypertrophic cardiomyopathy in a general population of young adults. Circulation.

[2] Bazan S.G.Z., Oliveira G.O., Silveira C.F.S.M.P. (2020). Hypertrophic cardiomyopathy: a review. Arq Bras Cardiol.

[3] Asil S., Kaya B., Canpolat U. (2018). Septal reduction therapy using nonalcohol agent in hypertrophic obstructive cardiomyopathy: Single center experience. Catheter Cardiovasc Interv.

[4] Vakrou S., Vlachopoulos C., Gatzoulis K.A. (2021). Risk stratification for primary prevention of sudden cardiac death in hypertrophic cardiomyopathy. Arq Bras Cardiol.

[5] Ommen S.R., Ho C.Y., Asif I.M. (2024). 2024 AHA/ACC/AMSSM/HRS/PACES/SCMR Guideline for the management of hypertrophic cardiomyopathy: a report of the American Heart Association/American College of Cardiology joint committee on clinical practice guidelines. J Am Coll Cardiol.

[6] Osman M., Kheiri B., Osman K. (2019). Alcohol septal ablation vs myectomy for symptomatic hypertrophic obstructive cardiomyopathy: systematic review and meta-analysis. Clin Cardiol.

[7] Sorajja P., Ommen S.R., Holmes D.R. (2008). Alcohol septal ablation versus surgical septal myectomy: comparison of effects on left ventricular outflow tract gradient and symptoms in hypertrophic obstructive cardiomyopathy. J Am Coll Cardiol.

[8] Veselka J., Krejčí J., Tomašov P. (2016). Long-term clinical outcome after alcohol septal ablation for obstructive hypertrophic cardiomyopathy: results from the euro-ASA registry. Eur Heart J.

[9] Li Y., Chen S.H., Guniganti R. (2022). Onyx embolization for dural arteriovenous fistulas: a multi-institutional study. J Neurointerv Surg.

[10] Souza A.L., Demuner P.F., Gardenghi G. (2025). Septal artery embolization with onyx® in hypertrophic cardiomyopathy: report of two cases. Arq Bras Cardiol.

